# Autophagy induction and CHOP under-expression promotes survival of fibroblasts from rheumatoid arthritis patients under endoplasmic reticulum stress

**DOI:** 10.1186/ar2921

**Published:** 2010-02-01

**Authors:** Yong-Joo Shin, Song-Hee Han, Do-Sung Kim, Geum-Hwa Lee, Wan-Hee Yoo, Yong-Mo Kang, Je-Yong Choi, Yong Chul Lee, Seoung Ju Park, Seul-Ki Jeong, Hyung-Tae Kim, Soo-Wan Chae, Hyun-Ja Jeong, Hyung-Ryong Kim, Han-Jung Chae

**Affiliations:** 1 Department of Rheumatology, Medical School, the Catholic University of Korea, Seoul, Republic of Korea, 150-713; 2 Department of Pharmacology and Cardiovascular Research Institute, Medical School, Chonbuk University, Jeonju, Chonbuk, Republic of Korea, 561-181; 3 Division of Rheumatology, Department of Internal Medicine, Medical School, Chonbuk University, Jeonju, Chonbuk, Republic of Korea, 561-181; 4 Division of Rheumatology, Department of Internal Medicine, Kyungpook University Hospital, Daegu, Republic of Korea, 561-181; 5 Department of Biochemistry and Cell Biology, School of Medicine, Kyungpook University, Daegu, Republic of Korea, 110-749; 6 Department of Internal Medicine, Medical School, Chonbuk Univ, Jeonju, Republic of Korea, 561-181; 7 Research Center for Pulmonary Disorders, Chonbuk Hospital, Jeonju, Republic of Korea, 561-181; 8 Department of Neurology, Medical School, Chonbuk University, Jeonju, Chonbuk, Republic of Korea, 561-181; 9 Department of Anatomy, Medical School, Chonbuk University, Jeonju, Chonbuk, Republic of Korea, 561-181; 10 Biochip Research Center, Hoseo University, Chungnam, Republic of Korea, 336-795; 11 Department of Dental Pharmacology, Dental School, Wonkwang University, Iksan, Chonbuk, Republic of Korea, 570-749

## Abstract

**Introduction:**

Synovial fibroblasts from rheumatoid arthritis show resistance to apoptotic stimuli, indicating they may be difficult to treat. To clearly understand these mechanisms of resistance, rheumatoid and osteoarthritis synovial fibroblasts (RASF and OASF) were exposed to endoplasmic reticulum (ER) stress such as thapsigargin, Ca^2+^-ATPase inhibitor.

**Methods:**

Fibroblasts were assessed microscopically for cell viability by trypan blue exclusion and for autophagic cells by LC-3II formation. Caspase-3 activity was measured as aminomethyl-coumarin (AMC) liberated from AC-DEVD-AMC. Immunoblotting was performed to compare protein expression in OASF and RASF.

**Results:**

ER stress caused cell death in OASF but not in RASF. Thapsigargin, a Ca^2+^-ATPase inhibitor, did not change the expression of GRP78, an ER chaperone in OASF and RASF, but induced another ER stress protein, CCAAT/enhancer binding protein (C/EBP) homologous protein (CHOP) differently, showing high levels in OASF and low levels in RASF. Thapsigargin increased the autophagy response in RASF, with autophagosome formation, beclin expression, and LC3-II conversion. Transfection with beclin siRNA inhibited autophagy and increased the susceptibility to ER stress-induced cell death. On the other hand, CHOP siRNA increased autophagy and improved cell survival, especially in RASF, indicating that CHOP is involved in regulation of autophagy and cell death, but that low expression of CHOP protects RASF from apoptosis.

**Conclusions:**

Autophagy induction and CHOP under-expression increases cell resistance against ER stress-induced cell death in fibroblasts from rheumatoid arthritis patients.

## Introduction

Rheumatoid arthritis (RA) is the most common inflammatory disorder of the joints. It is characterized by chronic inflammation, autoimmune phenomena and synovial hyperplasia, which lead to the progressive destruction of articular structures [1]. Alterations in synovial cell apoptosis, which regulate tissue composition and homeostasis, affect the pathogenesis of rheumatoid arthritis [2,3]. These changes lead to synovial cell activation and contribute to both chronic inflammation and hyperplasia. The resistance of rheumatoid arthritis synovial fibroblasts to apoptosis is closely linked to the progressive destruction of articular cartilage. However, the detailed mechanisms that prevent rheumatoid arthritis-associated cells from undergoing programmed cell death are unclear.

The endoplasmic reticulum (ER) plays an important role in secretory cells, including synovial fibroblasts. Adaptive responses to the accumulation of misfolded proteins in the ER (namely ER stress) provide protection from cell death, as gene transfer-mediated overexpression of GRP78 reduces cell death induced by oxidative stress and Ca^2+ ^disturbances [4]. Persistent, excessive ER stress triggers cell death [5,6] via the initiation of apoptosis and the induction of CHOP or by activation of caspase-12-dependent pathways [7,8]. CHOP mRNA is transcribed mainly during ER stress [8,9] and leads to apoptosis [10]. The ER stress also can contribute to autoimmune disease [11]. ER stress is studied in collagen-induced rheumatoid arthritis joints [12]. To study the role of ER stress in rheumatoid arthritis, we used synovial fibroblasts from rheumatoid arthritis patients, categorized according to ACR (American College of Rheumatology) classification criteria [13], to study apoptosis. In this study, ER stress response was examined in relation to the resistance characteristics in rheumatoid arthritis synovial fibroblasts (RASF).

Autophagy is implicated in various diseases, including cancer and neurodegenerative diseases [14-16]. During autophagy, a single-membrane structure (isolation membrane) surrounds a portion of the cytoplasm and organelles [14]. Autophagy can protect cells from ER stress-induced cell death [17]. Another explanation for apoptosis resistance in RASF could be that the unique cellular phenotype induced by autophagy protects against apoptotic stress. Here, we compare the response to ER stress and autophagy induction between synovial fibroblasts from rheumatoid arthritis and those from osteoarthritis.

## Materials and methods

### Cell cultures

Synovial fibroblasts were isolated from surgical samples from 13 rheumatoid arthritis and 8 osteoarthritis patients. Informed patient consents were obtained for isolation of fibroblasts. Cells were obtained by enzymatic digestion as described before [18]. Cells were grown in Dulbecco's modified Eagle's medium (DMEM) (Sigma-Aldrich, St. Louis, MO, USA) with 10% fetal calf serum (Gibco-BRL, Grand Island, NY, USA). The fibroblasts were cultured for six to eight passages. All studies were approved by the Chonbuk National Hospital ethics committee.

### Cell viability

Fibroblasts were assessed microscopically for dead cells by trypan blue exclusion. Cell viability was calculated by dividing the non-stained (viable) cell count by the total cell count. The number of cells was determined by averaging the number of cells in four squares and multiplying this average by a dilution factor.

### Measurement of autophagy

Autophagy was analyzed as described before [19]. Synovial fibroblasts from osteoarthritis and rheumatoid arthritis patients were plated at 2 × 10^5 ^on glass coverslips in six-well plates and cultured to 70% confluence. Cells were transfected with GFP-LC3 plasmid DNA (kindly provided by Dr. T. Yoshimori, Osaka University, Japan) for 16 h and then treated with thapsigargin or tunicamycin for various times. Transfection was performed using an Amaxa Nucleofector apparatus (Amaxa, Cologne, Germany). Five μg of plasmid DNA were mixed with 0.1 ml of cell suspension, transferred to a 2.0-mm electroporation cuvette, and transfected using an Amaxa Nucleofector apparatus (Amaxa, Cologne, Germany) according to the manufacturer's protocol. The DNA quantity, cell concentration and buffer volume were kept constant throughout the experiments. After electroporation, the cells were transferred immediately to 2.0 ml of complete medium and cultured in six-well plates at 37°C until needed. Microphotographs of GFP-LC3 fluorescence were obtained with a fluorescence microscope. The detection of punctuated staining of GFP-LC3 from diffuse staining indicated the formation of autophagosomes. The punctuated stained cells were compared to the total number of GFP-transfected cells to calculate percents.

### Determination of caspase-3 activity

Fibroblasts (3 × 10^6^) were washed with phosphate buffered saline (PBS) and incubated for 30 minutes on ice with 100 ml of lysis buffer (10 mM Tris-HCl, 10 mM NaH_2_PO_4_/NaHPO_4_, pH 7.5, 130 mM NaCl, 1% Triton1 X-100, and 10 mM sodium pyrophosphate). Cell lysates were spun down, supernatants were collected, and protein concentrations were determined using the BCA method. For each reaction, 30 μg of protein was added to 1 ml of freshly prepared protease assay buffer (20 mM HEPES pH 7.5, 10% glycerol, 2 mM dithiothreitol) containing 20 mM of AC-DEVD-AMC (Sigma-Aldrich). Reaction mixtures without cellular extracts were used as negative controls. Reaction mixtures were incubated for 1 h at 37°C and the aminomethyl-coumarin liberated from AC-DEVD-AMC was determined by spectrofluorometry (Hitachi F-2500, Hitachi, Tokyo, Japan) at 380 nm^excitation ^and 400 to 550 nm^emission^. Readings were corrected for background fluorescence.

### Western blotting

Western blotting was performed using the protocol described previously [19]. The total protein was resolved in pre-casted 4 to 12% SDS-PAGE gradient gels. Immunoblotting was performed using the indicated antibodies. ECL reagents (Amersham Biosciences, Piscataway, NJ, USA) were used to visualize signals.

### siRNA transfection

siRNAs were synthesized in duplex and purified using Bioneer technology (Daejon, South Korea). Double-stranded small interfering RNA (siRNA) targeting CHOP (SC-35437) was obtained from Santacruz company (Santa Cruz, California, USA) with control siRNA (SC-37007). For Beclin siRNA, 5'-CAGUUACAGAUGGAGCUAAtt-3' and for non-specific siRNA, 5'-CUUACGCUGAGUACUUCGAtt-3' were transfected into OASF and RASF using Amaxa Nucleofector (Amaxa, Gaithersburg, MD, USA). Briefly, confluent cells were trypsinized and resuspended in Amaxa Nucleofector solution at a density of 2 × 10^5 ^cells per 100 μl of solution, and each siRNA was added. Cells were transfected by electroporation using the A24 pulsing program.

### Statistical analysis

The data were analyzed by analysis of variance (ANOVA) in dose-response experiments, or by two-tailed Student's t-tests. A *P *value < 0.05 was considered significant. In each case, the statistical test used is indicated, and the number of experiments is stated in figure legends.

## Results

### Rheumatoid arthritis synovial fibroblasts (RASF) are resistant to ER stress-induced cell death

**Figure 1:**
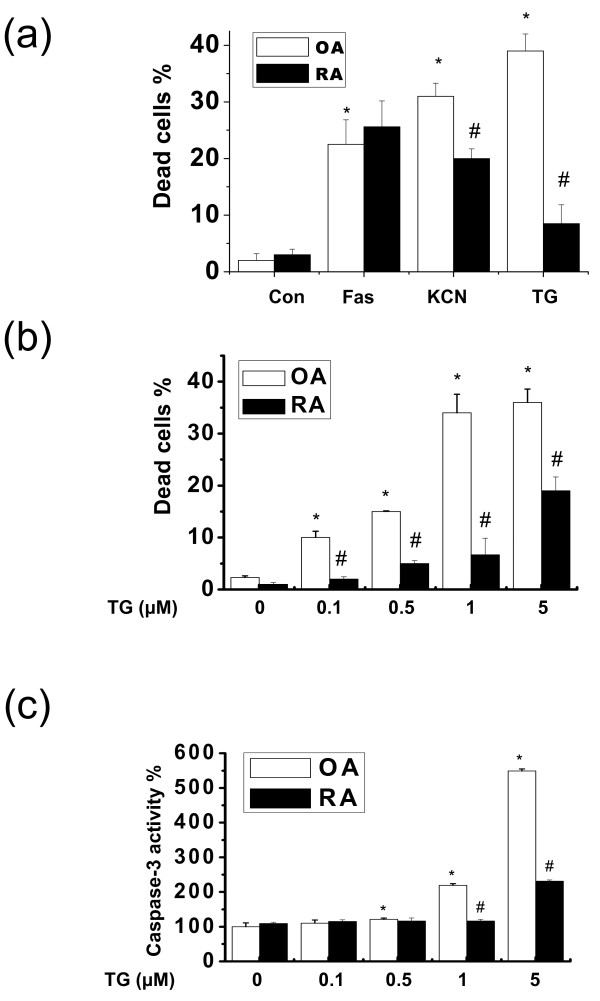
**Thapsigargin induces apoptosis in osteoarthritis synovial fibroblasts and rheumatoid arthritis synovial fibroblasts**. Osteoarthritis synovial fibroblasts and rheumatoid arthritis synovial fibroblasts (OASF and RASF, respectively) were treated with anti-Fas antibody (500 ng/ml) or KCN (1 mM) for 24 h, and thapsigargin (TG 5 μM) was also treated for 60 h. Dead cells were counted by the Trypan blue method **(a)**. Thapsigargin (0, 0.1, 0.5, 1 or 5 μM) was added to OASF and RASF for 60 h. Dead cells were counted by the Trypan blue method **(b) **and caspase-3 activity was measured **(c)**. *P < 0.05, versus non-treated OASF. ^#^*P *< 0.05, versus OASF with same treatments.

Decreased susceptibility to apoptosis in rheumatoid arthritis might contribute to resistance to anti-rheumatoid arthritis medications [20]. To confirm the characteristics of this resistance, OASF and RASF were exposed to apoptotic stimuli, namely anti-Fas antibody, KCN and thapsigargin. RASF showed a higher resistance to thapsigargin than to other stresses (Figure 1a). Thapsigargin is a Ca^2+ ^disturbance agent that leads to the accumulation of unfolded proteins in the ER [21]. We therefore questioned whether RASF resists apoptosis induced by excess unfolded proteins in the ER. We compared the dose-dependent sensitivities of RASF to thapsigargin with that of OASF. RASF were relatively resistant to ER stress but OASF were not (Figure 1b). ER stress also decreased caspase-3 activity, the executive caspase, in RASF more than in OASF (Figure 1c), showing ER stress-induced apoptosis is negatively regulated in RASF.

### The expression of ER stress protein CHOP is lower in RASF than in OASF

**Figure 2:**
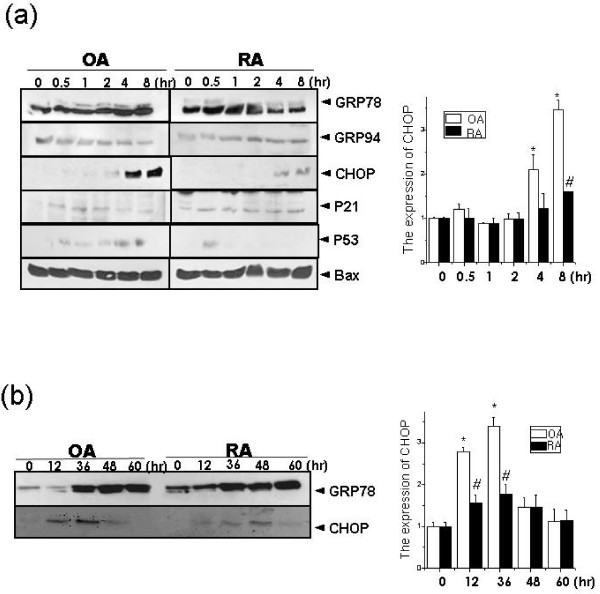
**CHOP expression is decreased in thapsigargin-treated RASF**. Thapsigargin (5 μM) was added for 0, 0.5, 1, 2, 4 and 8 h and SDS-PAGE and immunoblotting was performed with anti-GRP78, GRP94, CHOP, p21, p53 or Bax antibody (**a**: upper). The expression of GRP78 and CHOP was quantified (**a**: lower). OASF and RASF were treated with thapsigargin (5 μM) for 0, 12, 36, 48 and 60 h. After incubation, total protein was extracted. SDS-PAGE was performed and GRP78 and CHOP expression was analyzed (**b: **upper). The expression of CHOP was quantified (**b**: lower) *P < 0.05, versus non-treated OASF. ^#^*P *< 0.05, versus OASF with same treatments.

ER stress increases levels of stress proteins such as GRP78 or CHOP, as well as adaptation or cell death pathways [22]. In this study, we determined the expression levels of proteins involved in ER-stress-induced cell death in OASF and RASF. Intriguingly, the expression level of the pro-apoptotic protein, CHOP, was significantly decreased in RASF (Figure 2a). However, the expression of glucose response protein 78 (GRP78), also involved in ER stress responses, was similar in OASF and RASF. In addition, elongation initiation factor-1α (eIF-1α), the downstream protein of PERK, a PKR (RNA-dependent protein kinase)-like ER kinase that attenuates protein translation in response to ER stress, was similar in OASF and RASF (data not shown). When stress was prolonged more than 24 h, CHOP expression remained lower in RASF than OASF (Figure 2b). These results indicate that CHOP expression is regulated in RASF, which could explain resistance to ER stress-induced cell death.

### ER stress-induced autophagy is highly induced in RASF

**Figure 3:**
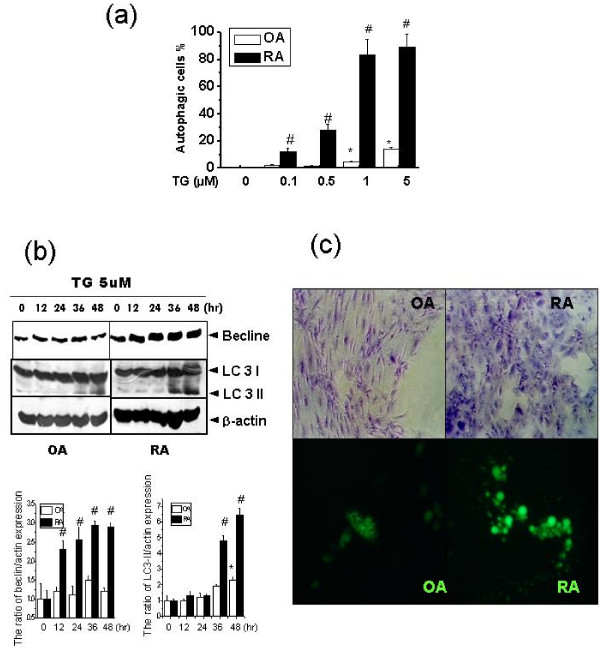
**Thapsigargin increases autophagy in RASF**. OASF and RASF were incubated with thapsigargin (0, 0.1, 0.5, 1 or 5 μM) for 60 h. Autophagic cell number was determined by autophagic vesicles. Data represent means ± S.E. (n = 4) **(a)**. Thapsigargin (5 μM) was added for 0, 12, 24, 36 or 48 h. After incubation, total protein was extracted. SDS-PAGE and immunoblotting were performed with anti-beclin, LC3 or actin antibody (**b: **upper). The expression of beclin and LC3-II was quantified, compared with the expression of actin (**b**: lower). OASF and RASF were treated with thapsigargin (5 μM) for 60 h. Crystal violet-stained cells are shown by light microscopy (upper panel) and GFP-LC3-transfected cells are shown by fluorescent microscopy (lower panel) **(c)**. (a) to (b): *P < 0.05, versus non-treated OASF. ^#^*P *< 0.05, versus OASF with same treatments.

When misfolded proteins accumulate in the ER, this stress activates the unfolded protein response (UPR) to induce expression of chaperones and proteins involved in the recovery process [23]. Under conditions of ER stress, pre-autophagosomal structures are assembled, and autophagosomal transport to vacuoles is stimulated [24]. In this study, we examined whether ER stress induces autophagy in either OASF or RASF. RASF showed high levels of autophagy at relatively low doses of thapsigargin (1 μM) (Figure 3a) and forms autophagosomes (Supplementary Figure 1). ER stress also increased the expression of beclin, an autophagy marker protein, and LC3-II more in RASF than in OASF (Figure 3b). When autophagy is induced, intra-lumenal LC3 is degraded by lysosomal proteases, forming an 18 kDa form (LC3-I) and subsequently being processed to a membrane-bound form (LC3-II, 16 kDa) [25]. To verify autophagy, we measured crystal violet-stained vacuoles under a light microscope (Figure 3c). A GFP-LC3 (LC3, mammalian homolog of yeast Atg8) fusion gene was transfected into OASF and RASF to measure changes in autophagosome numbers after treatment. The classical expression pattern of processed LC3-II was more evident in RASF, indicating autophagy vesicle formation. The expression was quantified in the lower panel of Figure 3c.

### A balance of autophagy and CHOP expression regulates cell death in RASF

**Figure 4:**
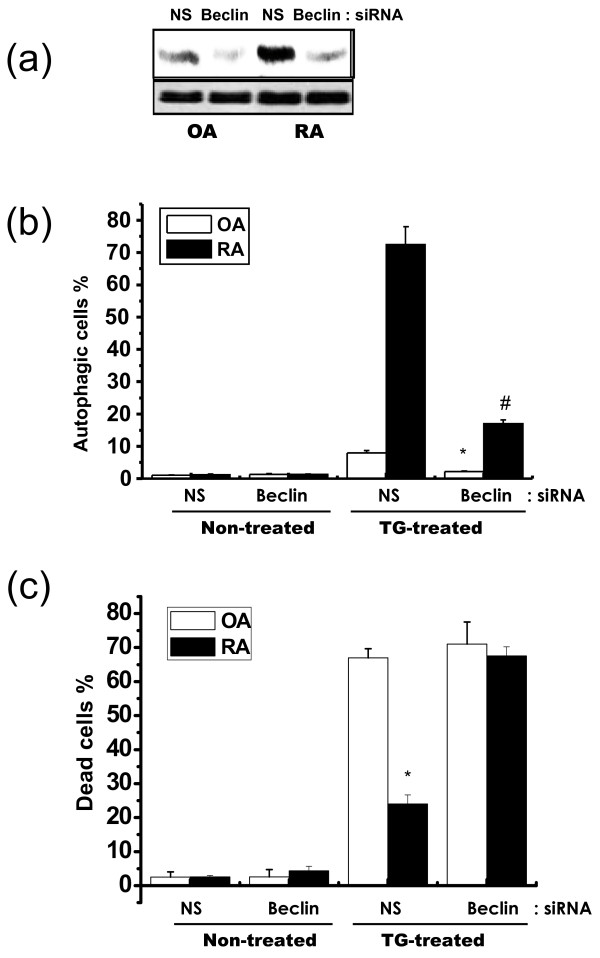
**Autophagy protects against ER stress in RASF**. OASF and RASF were transfected with non-specific or beclin siRNA, followed by thapsigargin treatment for 60 h. Non-specific and beclin siRNA were transfected into OASF and RASF. Sixteen hours later, total protein was extracted. SDS-PAGE and immunoblotting were performed with anti-beclin or actin antibody **(a)**. Autophagosomes **(b) **and dead cells **(c) **were counted as previously described. Data represent means ± S.E. (n = 6). *P < 0.05, versus non-specific siRNA-transfected OASF with thapsigargin. ^#^*P *< 0.05, versus beclin siRNA-transfected OASF with thapsigargin.

Autophagy is induced under ER stress conditions to protect against cell death [26,27]. In this study, we examined the role of ER stress-induced autophagy via knock-down of the autophagy marker, beclin. In OASF and RASF, transfection of beclin siRNA inhibited the expression of beclin, showing efficient transfection (Figure 4a). In RASF, the beclin siRNA decreased autophagy (Figure 4b) and increased cell death (Figure 4c). Beclin siRNA transfection did not affect cell death in OASF (Figure 4c). To understand the pathological meaning of autophagy, we tested its regulatory effect in RASF. In RASF, Ca^2+^-induced autophagy is regulated by hydroxychloroquine, a routinely used Disease-Modifying Anti-Rheumatic Drug (DMARD) that inhibits autophagy [28]. Hydroxychloroquine also increased susceptibility to cell death in thapsigargin-treated RASF (data not shown).

**Figure 5:**
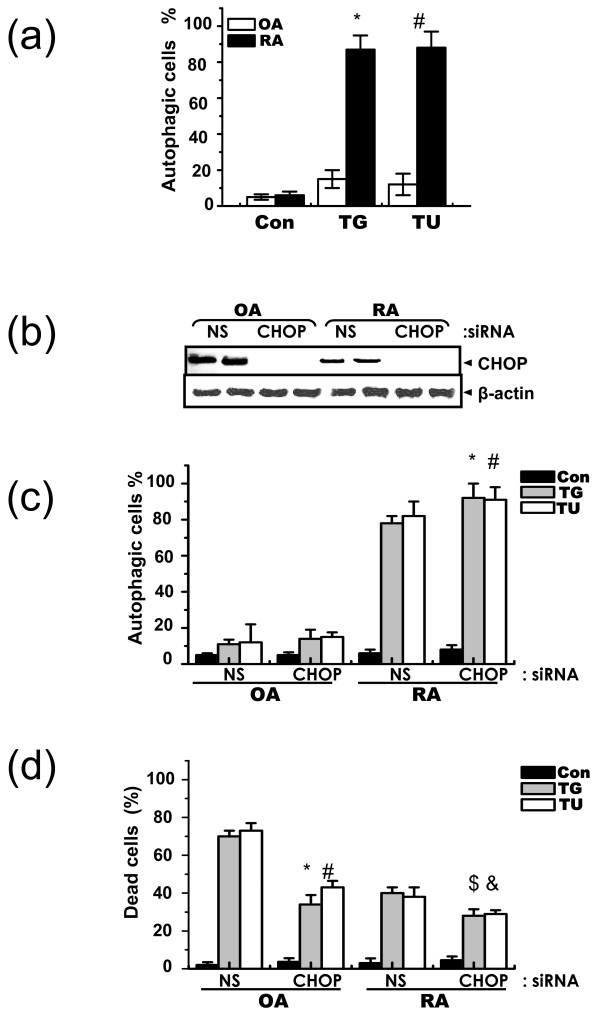
**CHOP plays an important role in autophagy in RASF and in cell death in OASF**. OASF and RASF were treated with thapsigargin (5 μM) or tunicamycin (5 μg/ml) for 60 h. Autophagy formation was measured as described in methods *P < 0.05, significantly different from thapsigargin-treated OASF, ^#^*P *< 0.05, significantly different from tunicamycin-treated OASF **(**a**)**. Non-specific or CHOP siRNA was transfected into OASF and RASF. After 16 h, cells were treated with thapsigargin (5 μM) or tunicamycin (5 μg/ml) for 4 h. Immunoblotting was performed with anti-CHOP or actin antibody **(**b**)**. Autophagy formation **(**c**)** and cell death **(**d**)** were measured. *P < 0.05, significantly different from non-specific siRNA-transfected OASF in the presence of thapsigargin. ^#^*P *< 0.05, significantly different from non-specific siRNA-transfected OASF in the presence of tunicamycin, ^$^*P *< 0.05, significantly different from non-specific siRNA-transfected RASF in the presence of thapsigargin. ^&^*P *< 0.05, significantly different from non-specific siRNA-transfected RASF in the presence of tunicamycin.

Autophagy plays an important role in the characteristics of RASF. In light of this, we examined other ER stress agents in OASF and RASF. First, we treated cells with tunicamycin, an N-acetyl glycosylation inhibitor. In RASF, tunicamycin increased autophagy in a similar manner as thapsigargin (Figure 5a). Using this model, we studied the effect of CHOP. First, CHOP siRNA was transfected into OASF and RASF. CHOP expression was barely detected in either OASF or RASF (Figure 2a). To show transfection efficiency, immunoblots were performed under ER stress-treated conditions. The siRNA knock-down approach successfully inhibited CHOP expression in both thapsigargin and tunicamycin-treated OASF and RASF (Figure 5b). CHOP inhibition significantly increased autophagy in RASF (Figure 5c) and increased cell viability (Figure 5d). Inhibition of CHOP increases cell viability in OASF, clearly showing the pro-apoptotic role of CHOP in OASF as well as in RASF (Figure 5d). These data suggest an inverse relation between CHOP expression and autophagy induction to increase cell resistance against ER stress in RASF.

## Discussion

The present study investigated the effects of ER stress on cell death in rheumatoid arthritis synovial fibroblasts (RASF). When exposed to ER stress, cell death and expression of the pro-apoptotic ER stress protein, CHOP, were lower in RASF than in OASF (Figure 1a and 1b). Furthermore, autophagy was significantly higher in RASF (Figure 3a, 3b, and 3c) than in OASF. Beclin siRNA transfection also showed that the formation of autophagosomes is related to the protective effect against ER stress in RASF (Figure 4b and 4c). CHOP siRNA protected cells from ER stress, showing that induction of CHOP explains cell death in RASF as well as in OASF (Figure 5d). In RASF, the knock-down of CHOP increased autophagy induction, which was related to cell protection (Figure 5c and 5d). ER stress in RASF showed autophagy and lower CHOP expression, increasing resistance to death.

Autophagy is a protective mechanism against apoptotic stimuli [26,29,30]. ER stress, which induces autophagy and apoptosis, is a pathological mechanism for disease [31-33]. GRP78, an ER stress protein, is associated with collagen-induced rheumatoid arthritis [34]. Normally, CHOP is ubiquitously expressed at very low levels [35], but is robustly expressed when perturbations induce stress [35], and CHOP^-/- ^cells are resistant to ER-stress-mediated apoptosis [36,37]. In OASF, CHOP expression was significantly increased at 2 h after treatment with thapsigargin, and reached a maximum after 8 h. In OASF, its expression was significantly increased at both 12 h and 36 h (Figure 2b), causing failure of the defense mechanisms and subsequent cell death.

To show direct evidence for the role of CHOP in ER stress-induced cell death, CHOP siRNA transfection was compared between OASF and RASF. As expected, the knock-down of CHOP increased cell viability, especially in OASF (Figure 4d). Because ER stress rarely affects autophagy in OASF, CHOP inhibition did not affect autophagy formation in OASF (Figure. 4c). These results are consistent with other studies that show CHOP as a pro-apoptotic protein [38,39]. In addition, CHOP expression after treatment with thapsigargin is lower in RASF than OASF, suggesting resistance against ER stress-induced cell death.

To explain the mechanism of the findings (that is, the increased autophagy in RASF), we compared the characteristics of OASF and RASF when exposed to ER stress. First, pro-inflammatory cytokines, including IL-6, were significantly higher in RASF. However, neutralizing antibodies for the cytokines did not affect autophagosome formation (data not shown). Second, there was no difference in intra-ER calcium between OASF and RASF when exposed to thapsigargin [40]. Therefore, the role of CHOP as a pro-apoptotic protein is more convincing than other possibilities. This is the first study on the induction of autophagy and ER stress that compares OASF and RASF. Increased autophagy induction and CHOP underexpression could explain the anti-apoptotic characteristics of RASF, at least when exposed to ER stress. An in depth study of CHOP will clarify the resistance to apoptosis in rheumatoid arthritis.

## Conclusions

RASF resists apoptosis following ER stress, such as Ca^2+ ^disturbances, by autophagy formation, which may contribute to resistance against rheumatoid arthritis treatments. A better understanding of the mechanisms contributing to apoptosis resistance through autophagy will provide better insight into the mechanisms of rheumatoid arthritis and help to identify targets for the development of novel, more effective and long-lasting therapies for the treatment of rheumatoid arthritis.

## Abbreviations

AMC: aminomethyl-coumarin; CHOP: CCAAT/enhancer binding protein (C/EBP) homologous protein; eIF-1: elongation initiation factor-1; ER: endoplasmic reticulum; GFP: green fluorescent protein; LC3: microtubule-associated protein 1 light chain 3; OASF: osteoarthritis synovial fibroblasts; PERK: a PKR (RNA-dependent protein kinase)-like ER kinase; RASF: Rheumatoid synovial fibroblast; UPR: unfolded protein response.

## Competing interests

The authors declare that they have no competing interests.

## Authors' contributions

YS participated in the design of the study and the experiments. SH performed autophagy experiments and autophagy mechanism studies. DK and GL carried out cell viability experiments. WY participated in the design of the study and provided fibroblasts. YK, JC, YL, SP, SJ, SC and HRK contributed to the experimental designs and the interpretation of the data. HTK performed electron microscopy experiments. HJ performed Western blotting experiments. HC supervised all of the experiments. All experiments were performed and supervised in HC's laboratory. All authors read and approved the final manuscript.

## Supplementary Material

Additional file 1 **Electron microscopy data**. Electron microscopy data from thapsigargin (1 μM)-treated OASF and RASF. Click here for file
