# Supplementary figures and images for: Autophagy induction and CHOP under-expression promotes survival of fibroblasts from rheumatoid arthritis patients under endoplasmic reticulum stress
Source: Arthritis Res Ther. 2010 Feb 1;12(1):R19. doi: 10.1186/ar2921 (PMC2875648; doi:10.1186/ar2921)

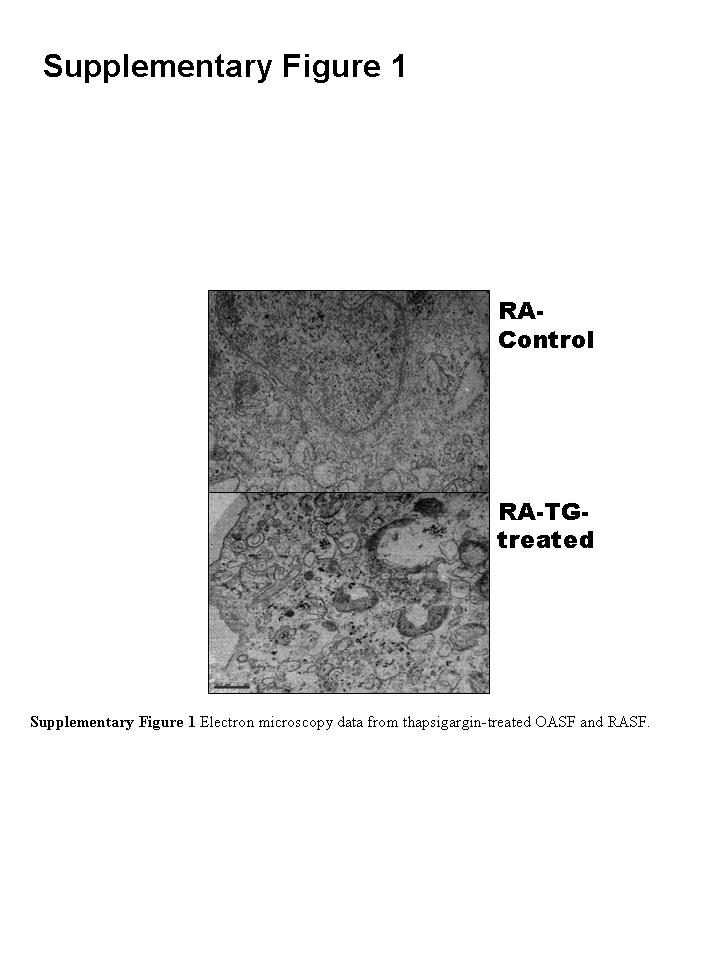

Supplement: Additional file 1 — Electron microscopy data. Electron microscopy data from thapsigargin (1 μM)-treated OASF and RASF. [file ar2921-S1.jpeg]
